# Comparison of the Automated OvaCyte Telenostic Faecal Analyser versus the McMaster and Mini-FLOTAC Techniques in the Estimation of Helminth Faecal Egg Counts in Equine

**DOI:** 10.3390/ani13243874

**Published:** 2023-12-16

**Authors:** Nagwa Elghryani, Conor McAloon, Craig Mincher, Trish McOwan, Theo de Waal

**Affiliations:** 1 Telenostic Limited, R95 WN20 Kilkenny, Ireland; craig@vemexis.com (C.M.); trish.mcowan@telenostic.com (T.M.); 2 Department of Biology, Faculty ofSciences-Qumnis, University of Benghazi, Benghazi 1308, Libya; 3 School of Veterinary Medicine, University College Dublin, D04 D6F6 Dublin, Ireland; conor.mcaloon@ucd.ie (C.M.); theo.dewaal@ucd.ie (T.d.W.)

**Keywords:** OvaCyte Telenostic, McMaster, Mini-FLOTAC, Equine helminths, Ireland

## Abstract

**Simple Summary:**

The faecal examination test is commonly employed for the identification and counting of parasite eggs in faecal samples. In this study, the OvaCyte Telenostic analyser (OCT) was used for detecting and counting helminth parasites in equine and compared with the McMaster and Mini-FLOTAC techniques. The prevalence of strongyle-type eggs was the highest of the detected helminth parasites. There was a strong correlation and agreement between the OCT and the other two counting techniques in this study. In terms of measuring the sensitivity and specificity, the OCT had the highest sensitivity for the detection of strongyles, *Parascaris* spp., and *Anoplocephala* spp., compared to the other two techniques.

**Abstract:**

Gastrointestinal helminth parasites continue to be a significant threat to the health of equine. OvaCyte Telenostic (OCT) (Telenostic Ltd., Kilkenny, Ireland) has developed an automated digital microscope utilising Artificial Intelligence to identify and count the clinically important helminth species in equine, bovine, and ovine host species. In this paper, the performance of the OCT analyser was evaluated for the detection and counting of equine helminth species parasites and its performance compared to the currently accepted benchmark methods of faecal egg counts being the McMaster and the Mini-FLOTAC techniques. A pairwise comparison of tests was assessed based on the correlation of egg counts and Cohen’s kappa agreement statistics for dichotomized outcomes. Bayesian latent class analysis was used to estimate the sensitivity and specificity of all three techniques in the absence of a gold standard for four helminth parasites (strongyles, *Anoplocephala* spp., *Parascaris* spp. and *Strongyloides westeri*). Based on the analysis of 783 equine faecal samples, we found a high level (ρ ≥ 0.94) of correlation between each pairwise comparison of techniques for strongyle egg counts. Cohen’s kappa agreement between techniques was high for strongyles and *S. westeri*, moderate for *Parascaris* spp., and low for *Anoplocephala* spp. All three techniques had a high sensitivity and specificity (>0.90) for strongyles. Across helminth parasites, the sensitivity of the OCT was the highest of the three techniques evaluated for strongyles (0.98 v 0.96 and 0.94), *Anoplocephala* spp. (0.86 v 0.44 and 0.46) and *Parascaris* spp. (0.96 v 0.83 and 0.96); but lowest for *S. westeri* (0.74 v 0.88 and 0.88), compared to McMaster and Mini-FLOTAC, respectively. In terms of specificity, OCT was the lowest in two species (*Parascaris* spp. 0.96, *Anoplocephala* spp. 0.95). In conclusion, OCT has a sensitivity and specificity statistically similar to both McMaster and Mini-FLOTAC, and had a higher correlation with Mini-FLOTAC. The OCT point of care faecal analyser offers improved workflow, test turn-around time and does not require trained laboratory personnel to operate or interpret the results

## 1. Introduction

Gastrointestinal helminths (GIH) are globally the most important cause of disease and production loss in grazing animals [1,2]. Many species of GIH (nematodes, cestodes, trematodes) at different stages of development are found to infect horses throughout their lives [3,4]. They cause inflammation, anaemia, colic, diarrhoea, and petechial haemorrhages as a result of adherence and penetration of the mucosa of the gastrointestinal tract. These parasites continue to be a significant threat to the health of horses and a leading impediment to successful horse rearing all over the world [5,6].

The large strongyles and small strongyles (cyathostomins) are considered to be the most prevalent and important helminths affecting the health of horses, particularly where management has been poor [6,7,8]. Several studies conducted between 2009 and 2021 have highlighted a high prevalence of strongyles with 39.5% to 94.6% reported [9,10,11,12,13].

*Strongyloides westeri* is the first naturally acquired nematode parasite that occurs in the small intestinal of foals. *S. westeri* is mainly found in young horses aged 4 to 8 months [14,15]. The egg count of this parasite is reported to significantly decrease at age 6-8 months and faecal egg counts were significantly associated with worm counts [14]. *S. westeri* is a rare cause of disease, however, studies found that, depending on parasite count, foals can develop acute diarrhoea [15,16,17]. The low prevalence of this parasite is documented by several research studies to range from 0.89% to 30% [9,11,15].

*Parascaris* spp. is a common nematode parasite that occurs in the small intestine of horses worldwide and is particularly important in young horses. *Parascaris* spp. is one of the rare equine nematodes that induces absolute acquired immunity, so this parasite is only of clinical significance in foals and yearlings. Clinical signs include impaction, catarrhal enteritis, severe diarrhoea, general malaise, and debility or even death [18,19,20,21,22].

*Anoplocephala* spp. is the most common tapeworm parasite of horses, which causes significant inflammation and damage of the mucosa at the region of the ileocecal junction. Massive infections may interfere with gut motility and increase the risk of ileocecal colic, diarrhoea, and weight loss, and can cause fatal intestinal blockage when a large number of tapeworms cluster in the ileocaecal area [23,24,25].

Detecting GIH disease hinges significantly on the diagnostic tools employed for identifying helminth infections. Regular faecal examinations play a vital role in recognizing and measuring parasite burdens, aiding in the diagnosis and control of GIH in horses. The accuracy and nature of diagnostic tools employed greatly influence the proper diagnosis and treatment of helminth infections. Regular faecal examinations are a common practice for pinpointing and gauging helminth infections, thus contributing to effective equine management [26]. Faecal egg counts (FEC) is the most widely used technique in both field and laboratory settings and is advocated by both the World Association for the Advancement of Veterinary Parasitology (WAAVP) and the Association of American Equine Practitioners in their guidelines [27,28]. They provide guidelines for both standard FEC for screening and targeted treatment, as well as faecal egg count reduction tests (FECRT) for evaluating the efficacy of anthelmintic drugs [29,30,31]. Currently, the McMaster (MCM) and Mini-FLOTAC (MF) techniques are the most frequently used methods based on the manual identification and counting of parasite eggs in faecal samples through microscopic examination [32]. These need to be performed by a trained technician in a laboratory environment. Microscopic identification of parasites has been the cornerstone of parasite diagnostics and research for decades [33,34]. However, the accuracy and usefulness of the microscopical laboratory diagnostic methods of faecal examination can be influenced by many factors, such as differences in methodology, personal experience, time spent on the identification and counting of parasite stages in the faecal of the animal of interest. Variability caused by these factors is increased by error due to operator fatigue when a large number of samples are examined [35,36].

The paucity of economical and rapid diagnostic tools useful for monitoring control programs in which large numbers of faecal samples need to be examined rapidly with a high level of accuracy, makes it necessary to implement more precise, accurate and rapid diagnostic techniques. To this end, automated systems to detect, identify and quantify parasite loads in prepared faecal samples have been proposed as a solution [37,38,39].

A range of parameters can be calculated to evaluate the performance of FEC techniques, including repeatability, reproducibility, accuracy, precision, and diagnostic sensitivity and specificity [40]. With respect to diagnostic sensitivity and specificity, a particular problem arises since the new diagnostic test to be evaluated must be compared with a gold standard. However, currently used tests are known to have imperfect test characteristics themselves and therefore cannot be considered true gold standards. Consequently, non-gold standard latent class models have been proposed [41], and have recently been used in order to estimate the diagnostic sensitivity and specificity of new diagnostic tests for the enumeration of equine FECs [42].

OvaCyte Telenostic (OCT) (Telenostic Ltd., Kilkenny, Ireland) has developed an automated digital microscope that utilises artificial intelligence to identify and count the clinically important helminth species in equine, bovine and ovine species. We have previously shown comparable performance of OCT to conventional methods for the detection and enumeration of strongyle eggs in bovine samples [37]. The purpose of this study was to compare the OCT performance with the currently accepted benchmark techniques, MCM and MF, in terms of test agreement and sensitivity and specificity using where appropriate, non-gold standard Bayesian latent class analysis, for detecting and counting the equine helminth infections.

## 2. Materials and Methods

### 2.1. Faecal Sample Collection 

Between August and November 2020 a total of 783 equine faecal samples were collected. Horse owners provided 354 samples from their own individual horses through an initiative by Horse Sport Ireland. The balance of 429 samples were obtained from individual horses that were presented to the University College Dublin (UCD) Veterinary Hospital and the clinical veterinary practice at the UCD Equine Centre. Horses were a mixture of different breeds, sexes and ages. Each faecal sample was placed into a labelled container on collection and sent immediately to the laboratory, where it was stored at 4°C upon arrival. All samples were processed within three days of collection.

### 2.2. Sample Preparation

A number of faecal balls from each sample were mixed to ensure equal distribution of parasite eggs. Faecal matter was added to a graduated measuring cylinder which contained 42 mL of saturated sodium chloride solution (NaCI, specific gravity 1.2). Faecal matter was added until a total volume of 45 mL was reached thereby representing 3 g of faecal material (1:14 dilution ratio). The faecal matter was well homogenised with the floatation fluid using a spatula. The suspension was then filtered through a tea strainer to remove the large debris, and then through a wire mesh filter (Endecott, London, UK: aperture 212 μm) and collected in a beaker clearly marked with the sample reference number. The prepared suspension (filtrate) was then used for each of the three FEC techniques, described below, and repeated for each sample.

### 2.3. Test Procedure 

FECs were carried out using a quantitative modified MCM technique with a detection limit of 50 eggs/g (epg) [43] and the MF technique with a detection limit of 7.5 epg [44]. Using a pasture pipette, the filtrate was loaded into both chambers of the MCM slide (Vetlab Supplies, Pulborough, UK; 0.15 mL per chamber, total of 0.3 mL), and the MF chambers (University of Naples Federico II, Italy, 1 mL per chamber, total of 2 mL). The OCT cassette (Telenostic Ltd., Kilkenny, Ireland) was filled with approximately 2 mL of filtrate with a syringe until the channel was full. The OCT technique has a detection limit of approximately 10 epg. Flotation times were 5 min for the MCM slide, 10 min for the MF slide and 7 min for the OCT cassette. All 3 of the above steps were performed on the same filtrate and at the same time (mixing the sample thoroughly each time). In the case of the OCT technique the cassette was placed onto the OCT analyser and the standard 7 min automated flotation time was controlled by the analyser. Microscopic helminth egg identification and counting of the MCM slide and the MF slide were performed at 10x magnification using an Olympus BX40F4 microscope (Mason Technology).

For the OCT technique, the OCT instrument scan sequence was automatically initiated after the 7 min flotation time, and a series of approximately 187 images were automatically taken through a 10x 25NA plan achromatic objective lens with 5 MP camera (OvaCyte analyser is manufactured by Telenostic Ltd., Kilkenny, Ireland). The images were uploaded into the cloud based object store where the Telenostic artificial intelligence algorithms identified and counted the strongyle eggs in each sample. Results were recorded as the number of eggs that the model identified and counted, the number of images used by the model, the multiplication factor and the resultant epg for each sample/scan.

### 2.4. Statistical Analysis

#### 2.4.1. Correlation and Agreement between Methods

For strongyle egg counts, OCT egg counts were compared to the two reference techniques (MCM & MF) separately using the winsorized correlation coefficient [45]. This method was chosen as a robust alternative to the standard Pearson correlation coefficient and to limit the impact of outliers on the estimate. Correlations were not calculated for the remaining parasite species since the prevalence of each of these parasites was low. Agreement between techniques was calculated separately for each of the parasite species. Outcomes were dichotomised using clinical significant treatment thresholds: >200 epg for strongyles, ≥2000 for *Strongyloides westerii*, and ≥ 1 epg for *Anoplocephala* spp.and *Parascaris* spp. Cohen’s Kappa statistic was calculated separately between each pairwise comparison of the three techniques to evaluate the agreement between each test pair, whilst accounting for agreement due to chance. All analyses were conducted in R (R-studio Version 2022.07.1) [46]. Correlation coefficients were calculated using the “correlation” package [47] and Cohen’s kappa statistics using the “psych” package [48].

#### 2.4.2. Estimation of Sensitivity and Specificity

Sensitivity (Se) and specificity (Sp) were estimated using Bayesian Latent Class Analyses (BCLA). For each parasite, a 3-test, 1-population model was constructed to estimate 7 parameters: the sensitivity and specificity of each of the three techniques, and the true prevalence in the sampled population. Tests were assumed to be conditionally independent, and we assumed that the test results from each of the three techniques followed a multinomial sampling distribution:

y~Multinomial(n, (p_111_, p_100_, p_010_, p_001_, p_110_, p_101_, p_011_, p_000_))

With the multinomial cell probabilities defined as:

p_111_ = π × Se_OCT_Se_MCM_Se_MF_ + (1 − π) × (1 − Sp_OCT_)(1 − Sp_MCM_)(1 − Sp_MF_)

p_100_ = π × Se_OCT_(1 − Se_MCM_)(1 − Se_MF_) + (1 − π) × (1 − Sp_OCT_)Sp_MCM_Sp_MF_

p_010_ = π × (1 − Se_OCT_)Se_MCM_(1 − Se_MF_) + (1 − π) × Sp_OVT_(1 − Sp_MCM_)Sp_MF_

p_001_ = π × (1 − Se_OCT_)(1 − Se_MCM_)Se_MF_+ (1 − π) × Sp_OCT_Sp_MCM_(1 − Sp_MF_)

p_110_ = π × Se_OCT_Se_MCM_(1 − Se_MF_) + (1 − π) × (1 − Sp_OCT_)(1 − Sp_MCM_)Sp_MF_

p_101_ = π × Se_OCT_(1 − Se_MCM_)Se_MF_+ (1 − π) × (1 − Sp_OVT_)Sp_MCM_(1 − Sp_MF_)

p_101_ = π × (1 − Se_OCT_)Se_MCM_Se_MF_+ (1 − π) × Sp_OCT_(1 − Sp_MCM_)(1 − Sp_MF_)

p_000_ = π × (1 − Se_OCT_)(1 − Se_MCM_)(1 − Se_MF_) + (1 − π) × Sp_OCT_Sp_MCM_Sp_MF_

Test characteristics, Se and Sp, for each of the 3 tests, and true prevalence, π, were modelled as beta distributions. Uniform, Beta (1,1) distributions were used as priors for each of the sensitivity parameters, and for the true prevalence parameter. Specificity of each test was assumed to be greater than 80% with a 95% probability and with a mode at 0.99; therefore, a Beta (14.5, 1.14) distribution was used as the prior for the specificity for all three techniques. Convergence was assessed by inspection of the trace plots and by initiating two chains from dispersed starting values. The first 10,000 iterations were discarded as burn-in and a further 30,000 iterations used for posterior inference. Models were fitted using JAGS [49] using the “rjags” package in R [50].

## 3. Results

### 3.1. Overall Infection Distribution and Prevalence

MF, being a well-documented, accurate and precise test was used to evaluate the overall apparent prevalence of each of the four parasite species. In total, 531 (67.81%) out of the 783 horses were positive for at least one GIH.

A summary of the apparent prevalence of GIH species in 783 equine faecal samples was examined using the MF method, as shown in Table 1 and Table S1. 

### 3.2. Egg Count Comparisons Using the Three Different Techniques

**Figure 1:**
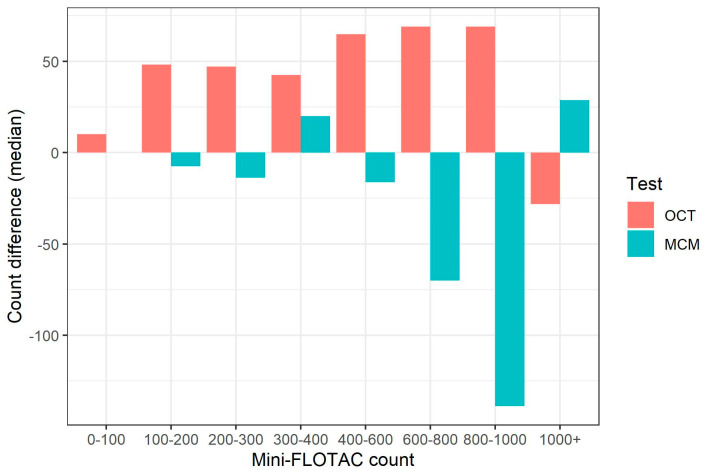
Median count difference between OCT and MCM compared to MF at different MF count ranges.

For strongyle eggs, count differences between each of the three techniques differed according to the degree of egg shedding. Figure 1 shows the median difference in egg counts between each of the three techniques according to a number of epg bands measured by MF. Compared to MF, OCT strongyle epgs were generally higher, except for high counts (>1000 epg) where OCT showed slightly lower counts than MF. In contrast, MCM counts tended to be lower than those returned by MF, apart from samples with egg counts between 300–400 and >1000 epg.

### 3.3. Level of Agreement between the Three Techniques

**Figure 2:**
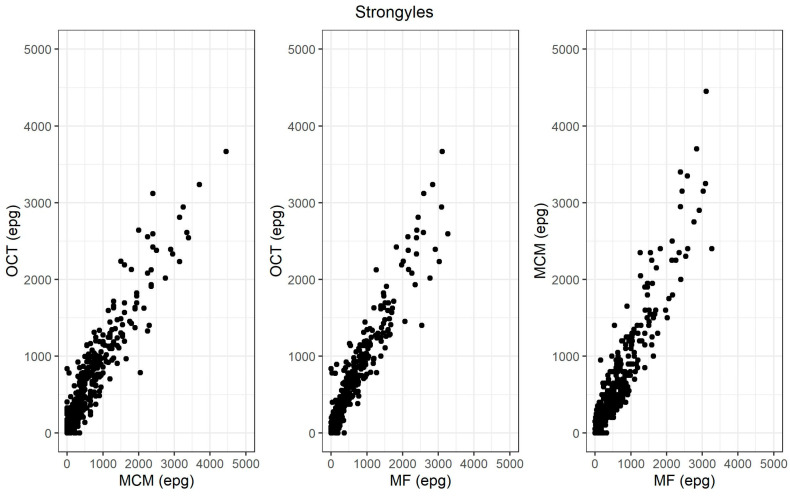
Scatter plot of the correlation between OCT, MCM and MF strongyle egg counts (eggs per gram).

The comparison of OCT with the two reference techniques for strongyle egg counts showed a higher correlation with MF (0.96, 95% CI = 0.96–0.97) than for MCM (0.94, 95% CI = 0.93–0.95) (Table 2) (Figure 2).

The Cohen’s Kappa agreement between techniques is shown in Table 3. The agreement between OCT and the other two techniques was high for strongyle epgs (0.87 with MF and 0.85 with MCM) and *Strongyloides westeri* (0.80 with both MF and MCM). However, there was poor agreement for *Anoplocephala* spp. and *Parascaris* spp. for each of the three pairwise comparisons.

### 3.4. Sensitivity and Specificity Estimation

Results of the Bayesian latent class analysis (BCLA) model posterior estimates of Se and Sp for each of the three techniques across each parasite species are shown in Table 4. For strongyles, all techniques had high (>90%) Se and Sp. However, the Se was the highest for OCT and Lowestr for MF. For *Parascaris* spp. Se was the highest for both MF and OCT (0.96, 0.85–1.00), and the lowest for MCM, whilst Sp was the highest for MCM and the lowest for MF. For *Strongyloides westeri,* Se was the highest for MCM and the lowest for OCT, whilst Sp was very high (>0.998) for all three techniques. Finally, *Anoplocephala* spp., Se was the highest for OCT and the lowest for MCM, whilst Sp was the highest for MCM and the lowest for OCT.

## 4. Discussion

FECs are a widely used method for detecting gastrointestinal parasites. The MCM and MF techniques are commonly employed for the manual identification and counting of parasite eggs in faecal samples. In this study, OCT was used for detecting and counting GIH in faecal samples and compared with the two commonly used techniques (MCM and MF). Overall, we found high levels of agreement between the three techniques evaluated and similar sensitivities and specificities.

We found that the prevalence of strongyle-type eggs were the highest of the detected helminth parasites evaluated in the study. This agrees with a previous study by Elghryani et al. [51], where the prevalence of strongyle infections in horses on Irish farms was 52.40% based on samples from 2700 horses between 2015 to 2021. This observation is also in agreement with international studies reporting a high prevalence of equine strongyles in Europe, the USA, and African countries [33,44,52,53,54].

Strongyle parasites are considered the most problematic group of helminths and the main targets of parasite control strategies due to their wide distribution and widespread anthelmintic resistance [6,55,56]. FECs for strongyles are already broadly used in the targeted treatment of horses [27,57,58,59], therefore, for accurate identification of treatment candidates, reliable egg counts are essential. In our study, the number of horses diagnosed as positive for strongyles differed between the three techniques with 72% of horses testing positive based on OCT and only 50% and 62% based on MCM and MF, respectively. The lower detection limits for both OCT and MF may explain why more horses tested positive for at least one egg in these two techniques. Strongyle egg counts were numerically higher when evaluated by OCT compared to both the other techniques, except in very high counts (>1000 epg) (Figure 1). At these high counts, MCM returned higher egg counts than both OCT and MF. The higher multiplication factor used in MCM is a potential reason for this. However, this finding is unlikely to have major clinical significance since the results are ultimately dichotomised according to established clinical thresholds, therefore differences between counts well in excess of this count are unlikely to impact clinical decision-making. Importantly, the Se of OCT was higher than MF and MCM based on a clinical significance threshold, indicating that OCT detects a higher proportion of animals requiring treatment. In contrast, Sp of OCT was lower than that for MF, but higher than MCM, although these differences were not significant. In practice this means that slightly more animals would likely be treated (2.3%) based on the 200 epg threshold when testing by OCT compared to MF, although it is worth noting that the Sp estimates for both tests were quite high, 0.962 and 0.985 for MF and OCT, respectively, and is unlikely to be of any clinical significance. Our finding of higher Se of MF than MCM for detecting the GIH is in agreement with existing studies [33,44,52,53,54], and is likely due to the differences in detection limits between the techniques, where the minimum detection limit is 7.5 epg with the MF compared to 50 epg with the MCM. Taken together, these findings suggest that OCT is at least as accurate as existing reference techniques for detection and enumeration of strongyle eggs in equines.

For the remaining parasites, there was fair to moderate agreement between pairwise comparisons of tests for *Anoplocephal* spp. and *Parascaris* spp. except in the comparison between MF and MCM where good agreement was observed. This finding highlights the limitation in using agreement tests and pseudo-gold standard comparisons to evaluate novel diagnostic tests. If a novel diagnostic test exhibits better test characteristics than existing reference tests, this will often manifest as poor agreement and poor Se/Sp when those existing tests are used as a reference standard [60]. In contrast, the non-gold standard BLCA approach taken in this study reveals that the Se of OCT for the detection of *Anoplocephal* spp. is likely to be much higher than that for the comparator techniques, indicating that a higher proportion of horses requiring treatment are detected using this test. Finally, there was good/strong agreement between all three techniques for the detection of *Strongyloides westeri*, along with high Se for all three tests, and very high Sp estimates.

There were several limitations with this study. Whilst the use of clinical samples improves the external validity of our results, the apparent and estimated true prevalence of three out of the four parasite species evaluated was low (<5%). The impact of these low prevalences is reflected in the wide probability intervals for the Se estimates, particularly for *S. westeri* and *Anoplocephal* spp. In contrast, the Sp estimates for these species were relatively precise as a result of the larger proportion of negative samples. The estimates for these samples could be improved with additional samples or with samples with a higher prevalence, although this may impact the external validity of the results depending on the population from which they are sourced.

## 5. Conclusions

The OvaCyte (Telenostic Ltd., Kilkenny, Ireland) automated faecal technique has a sensitivity and specificity statistically similar to both McMaster and Mini-FLOTAC, and had a higher correlation with Mini-FLOTAC. It is a viable alternative to the currently used benchmark FEC techniques (McMaster and Mini-FLOTAC) for the detection of the common GIH in horses. The OCT point of care faecal analyser offers improved workflow, test turn-around time and does not require trained laboratory personnel to operate or interpret the results.

## Figures and Tables

**Table 1:** Apparent prevalence of helminth parasites in 783 equine faecal samples tested using the MF technique.

Parasite	Positive at Least 1 egg (%)	>100 epg (%)	>500 epg (%)	>1000 epg (%)
Strongyles	486 (62.1)	319 (40.7)	163(20.8)	76 (9.7)
*Parascaris* spp.	27 (3.4)	13 (1.7)	4 (0.4)	3 (0.4)
*Strongyloides westeri*	12 (1.5)	12(1.5)	8 (1.0)	5 (0.6)
*Anoplocephala* spp.	6 (0.8)	1 (0.1)	0 (0.0)	0 (0.0)

**Table 2:** Correlation between OCT, and MCM and MF for strongyle egg counts.

Parasite	Correlation (OCT × MCM)	Correlation (OCT × MF)	Correlation (MF × MCM)
Strongyles	0.94 (0.93, 0.95)	0.96 (0.96, 0.97)	0.95 (0.94, 0.96)

**Table 3:** Cohen’s Kappa agreement between OCT and MCM and MF for each helminth parasite for dichomotomised test outcomes.

Parasite	Test Pair Comparison: OCT × MCM	Test Pair Comparison: OCT × MF	Test Pair Comparison: MF × MCM
Strongyles	0.85 (0.81, 0.89)	0.87 (0.83, 0.91)	0.85 (0.81, 0.89)
*Anoplocephala* spp.	0.17 (0.03, 0.32)	0.16 (0.02, 0.31)	0.40 (0.01, 0.78)
*Parascaris* spp.	0.50 (0.36, 0.65)	0.52 (0.38, 0.65)	0.79 (0.66, 0.93)
*Strongyloides westeri*	0.80 (0.53, 1.00)	0.80 (0.53, 1.00)	1.00 (1.00, 1.00)

**Table 4:** Posterior estimates and 95% posterior credible intervals (95% CI) of prevalence and test characteristics from Bayesian Latent Class Analysis.

Parasite	Estimated True Prevalence	Test	Sensitivity	Specificity
Strongyles	0.350 (0.317, 0.384)	MCM	0.955 (0.927, 0.978)	0.961 (0.927, 0.978)
		MF	0.938 (0.907, 0.967)	0.985 (0.972, 0.995)
		OCT	0.978 (0.957, 0.994)	0.962 (0.944, 0.978)
*Parascaris.* spp.	0.028 (0.017, 0.040)	MCM	0.833 (0.654, 0.972)	0.999 (0.996, 1.000)
		MF	0.963 (0.849, 1.000)	0.991 (0.983, 0.997)
		OCT	0.963 (0.849, 1.000)	0.960 (0.946, 0.973)
*Strongyloides westeri*	0.007 (0.002, 0.014)	MCM	0.880 (0.578, 1.000)	0.999 (0.996, 1.000)
		MF	0.879 (0.578, 1.000)	0.999 (0.996, 1.000)
		OCT	0.735 (0.408, 0.983)	0.998 (0.994, 1.000)
*Anoplocephala* spp.	0.014 (0.003, 0.031)	MCM	0.442 (0.092, 0.826)	0.999 (0.995, 1.000)
		MF	0.460 (0.108, 0.830)	0.997 (0.992, 1.000)
		OCT	0.869 (0.572, 1.000)	0.959 (0.941, 0.978)

## Data Availability

The data presented in this study are available on request from the corresponding author. The data is not publicly available.

## Supplementary Materials

The following supporting information can be downloaded at: https://www.mdpi.com/article/10.3390/ani13243874/s1, Table S1: Apparent prevalence of helminth species in 783 equine faecal samples tested using McMaster and OvCyte Telenostic techniques.
